# Impact of Regulatory Changes on Innovations in the Medical Device Industry: Comment on "Clinical Decision Support and New Regulatory Frameworks for Medical Devices: Are We Ready for It? - A Viewpoint Paper"

**DOI:** 10.34172/ijhpm.2022.7262

**Published:** 2022-06-15

**Authors:** Petra Maresova

**Affiliations:** Department of Economics, Faculty of Informatics and Management, University of Hradec Kralove, Hradec Kralove, Czech Republic.

**Keywords:** Innovation, Regulatory, Medical Device Industry

## Abstract

Economic regulation is an instrument of the state or other institutions to correct market failures, rectify the business environment, or protect consumers. Regulation can be a major driver of innovation, and it has proven to be so in the past. On the other hand, there are also documented cases of ineffective regulation due to information delays or shortcomings in government decision-making. The complexity of the impact of regulatory changes on innovation can currently be observed in the medical device market in Europe. Regulation (EU) 2017/745 whose main idea is to ensure greater safety and health protection for consumers, is a challenge for originator, manufacturer, mostly small and medium-sized enterprises. The regulation is associated with an increase in the cost of developing and maintaining the product on the market. We can now gradually begin to analyze whether it can be ranked among those that have become drivers of innovation.

## Introduction

 Innovation is currently the engine of both developed and developing economies.^1^ Decisions concerning new products are key to the growth and prosperity of any company, especially in the rapidly changing medical device market. Companies need innovations to succeed, but developing new products and processes is risky and usually more resource intensive compared to some other sectors of the economy.

 Economic regulation is an instrument of the state or other institutions to correct market failures, rectify the business environment, or protect consumers. Regulation can be a major driver of innovation, and it has proven to be so in the past. Examples include the environmental impact of regulation that started in the 1870s and is associated with the growth of the competitiveness of manufacturing companies in the United States or the regulation in the pharmaceutical industry that has proved to exert a positive influence on innovation in the United Kingdom in the twentieth century. On the other hand, there are also documented cases of ineffective regulation due to information delays or shortcomings in government decision-making.

 The link between regulation and innovation is complex, which makes it difficult to detect any direct causality. The complexity of the relationship between regulation and innovation stems from the fact that changes in the regulatory framework do not always trigger immediate and direct changes in innovation. Such changes in innovation sometimes occur indirectly, following changes in competition, in investment decisions, or other changes in corporate decision-making and management. According to the Porter hypothesis,^2^ which refers in particular to environmental regulation, strict regulation can dramatically stimulate innovation by replacing dominant technologies with new companies or new market players. Bonnin Roca and O’Sullivan^3^ note that regulatory bodies in the medical device sector focus on ensuring patient safety rather than fostering innovation. This influences variables such as costs, product availability, or financing options.^4^

 The complexity of the impact of regulatory changes on innovation can currently be observed in the medical device market in Europe. Regulation (EU) 2017/745 of the European Parliament and of the Council, on Medical Devices (MDR), which has been in force following a delay due to the coronavirus disease 2019 (COVID-19) pandemic since May 26, 2021, and whose main idea is to ensure greater safety and health protection for consumers, is a challenge for small and medium-sized enterprises in this sector. We can now gradually begin to analyse the impact of the regulation on companies and see whether it can be ranked among those that have become drivers of innovation. Regarding the impact of this regulation, the following two questions must be answered:

- How does a company on the medical device market assess investment in the development of a new product with regard to MDR 2017/745?
- What is the expected impact of MDR 2017/745 on the medical device sector and on the innovation activity of companies in this sector?

For the purpose of the present research, the medical device industry refers mainly to manufacturers of medical devices, including medical device software. Importers and distributors of medical devices are also included, but they are not affected by the new regulation so strongly.

## Companies’ Views on New Product Development

 Although it is not possible at this time to clearly assess the impact of the new regulation on the medical device sector due to its postponement and subsequently its short period of validity, partial conclusions from the current situation can be drawn and possible future developments discussed.

 It is clear that the aim of the MDR 2017/745 regulation is to introduce and implement comprehensive, in some respects stricter requirements for the placement of medical devices on the market and for their distribution within the European Union, with the main goal to increase clinical safety and improve the traceability of all medical devices. It is therefore a matter of protecting the health of patients and ensuring greater safety. The regulation also introduces the new central European Database on Medical Devices, which will list all medical devices placed on the European Union market as well as all actors in the distribution chain, including manufacturers, authorized representatives, importers, and distributors.

 This regulation, which replaces the previous directive and any individual national legislation, is similar to the regulation of the medical device industry in the United States, where the Food and Drug Administration is the regulatory body. In the United States, the medical device market is larger than in Europe, counting several times more companies, and the leaders of many innovative companies agree that regulation is necessary but also that current regulations must flexibly respond to changes in research and development, if regulation is to become a driver of innovation.^5^

 The medical device sector in Europe is composed mainly of small and medium-sized companies; even micro-enterprises are no rarity. In the day-to-day operation of many companies, it is common that there are no clear records and methodology of medical device development, there is no systematic monitoring of costs associated with repetitive activities, and there are other shortcomings as well.^6^ Regarding the attitudes of the companies themselves^7^ there is a clear concern about possible future developments, concerns about additional costs, dissatisfaction with the requirements for placing the product on the market in the European Union, and even thoughts about leaving the market and focusing on products other than medical devices. Large, strong companies tend to adapt, accepting the changes and concentrating their efforts on fulfilling the new requirements.

 It can be said that the conditions in some European Union countries do not correspond to the new regulation, especially in terms of notified bodies, since there is a shortage of notified bodies within the European Union,^8^ and some countries even lack a notified body entirely.^9^ The notified body is responsible for the medical device and is required to confirm the compliance of the product with the new, stricter safety requirements. Van Laere et al^10^ states that, for example, software vendors in countries without a notified body are forced to use notified bodies located in another country and may not be able to apply in their own language. This also implies that the number of applications will be larger for the existing notified bodies, leading to a greater workload and potentially delayed market access to new technologies. As to the readiness of companies for the regulatory change, a certain threat is represented by the approval process, its length, and its availability.

 Although the case study^11^ works with data from only one small company, the company model clearly shows that the way to future development and operation under new certification costs and new clinical trial responsibilities is to invest in new product development and enter new markets. Only under these conditions and with increased efforts is it possible to achieve a positive economic result. Another study, modelling the use of several modern technologies for providing care to the elderly,^12^ confirms that innovative solutions are the right way in terms of societal impacts as well. New solutions and their implementation help save labor time, which is in short supply in developed countries.^13^

## Innovation Activity of the Sector

 It is now clear that companies are making increased efforts to obtain a certification for their existing products. This fact resulted, for example, from a questionnaire survey conducted among producers, importers and distributors in the Czech Republic, which took place in the autumn of 2021 and was managed by University of Hradec Kralove.

 They are investing and further anticipating increased investments in the development of new medical devices, and they do not tend to see regulation as a stimulus for innovation. The situation will become clearer over the next few years, at which point the regulation could become an incentive for innovation under certain conditions. One of the factors could be the impact of higher costs on the price of the product. If the companies are forced to increase the product price due to the new legislative requirements, they will likely look to innovate the product itself or the associated services, so as to make the price increase acceptable to users. New, safer medical devices will have a competitive advantage over products from countries where the conditions for product development are not so strict (China, India). This can therefore lead to a development of the medical device industry.

 It will take several more years before it is possible to evaluate the impact of MDR 2017/745 on the medical device sector and its innovation activity, using the economic methods and indicators that have been used in the past to assess the impact of regulation.^5,14^

 The evaluation should also take into account the fact that MDR 2017/745 can be perceived as the first step, which will be followed by the Regulation on In Vitro Diagnostic Medical Devices (IVDR) 2017/746, where further significant impacts can be expected.

 Further amendments and explanatory documents to the MDR 2012/745 legislation may be needed in the near future, called for by the rapid development of innovations, including medical devices and the example of artificial intelligence algorithms. Authorities responsible for outlining and interpreting regulations should be open to cooperation and discussion with stakeholders when it comes to preparing additional explanatory documents. Research and marketing of innovative medical devices are developing fast, taking over the development of evaluation methods and processes. Cooperation among all stakeholders, particularly a cooperation between the regulatory organs and the business community, is necessary to achieve maximum clarity and effectivity of the regulation.

###  Measured Features 

 A more detailed view of the possibilities of measuring and monitoring the impact of legislation on companies is provided, for example, in Maresova et al,^11^ which clearly defines the individual phases of medical device development^6^ and the related parameters for evaluating the investment effectivity with respect to the changes in legislation. An innovative experimental approach^11^ proposes a combined dynamic model based on the existing company model on one hand and the medical device business model on the other hand. The resulting new model is designed to answer the following key questions: How will the new legislation impact the company’s economic performance? What is the break-even point for the company under the new legislation? How much will the company have to increase the product price to cover the increased costs of the new legislation? Or instead, how much will the company have to increase its market share to keep the current product price while at the same time covering the increased costs?

 Figure illustrates the dependencies between new medical device development and the company cash flow and market performance. A new medical device under the new legislation is developed in six phases, starting with initiation, concept development and product design, following with production and final verification, and ending with market placement. This process is however highly resource-intensive, and should the new device development fail at any point, the company will struggle with economic losses, leading to a drop in performance and possibly a threat to the company’s very existence. In contrast, with a successful development and market placement of a new product, the company increases its portfolio quality, decreases its product obsolescence, and ultimately boosts its economic performance. A new product may respond to the needs of a new group of patients, possibly leading to increasing the company’s market share, which is essential for a sustainable company growth.

**Figure:**
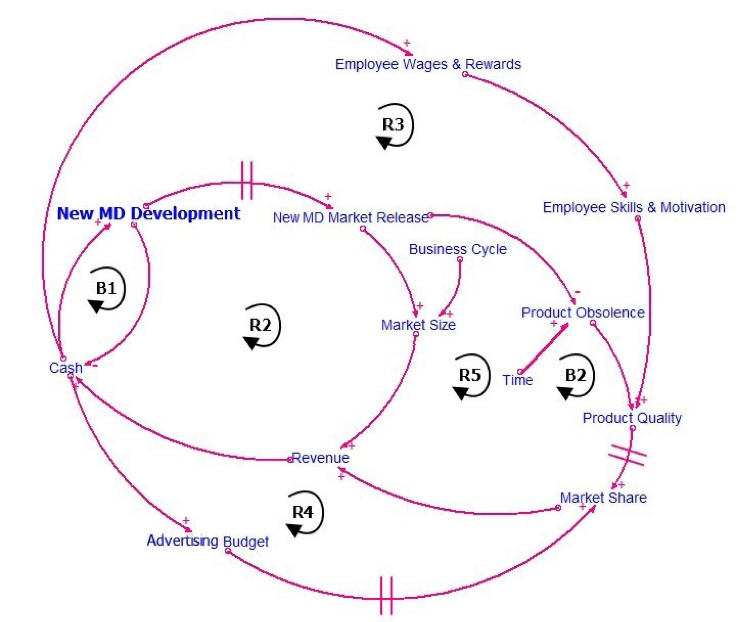
Causal Loop Diagram for MD Development and its Impact on the Company’s Cash Flow and Market Performance. Abbreviation: MD, Medical Devices. Source: Adapted from Maresova et al.^12^

 Each part of the casual loop diagram in Figure is described in detail. For example, in terms of the market, the increase or decrease of the company’s market share is modeled by comparing the relative product value with the product price. The market size can be regarded as a fixed parameter because it is limited by legislative regulation.

 The customer gain is determined by:


$$\Delta M_{+}=c\left(1-M\right)\left(\left(q-1\right)+m\delta^{\left(1-q\right)}\right),$$

capped so that the updated value of M does not exceed one, where *m = μa⁄d*, and other variables:*Market_Share* (M), *Customer_Awareness* (c), *Diminishing_Returns_Coefficient* (δ), *Marketing* (m), *Marketing_Effectiveness* (*μ*).

 Similarly, in terms of the quality, the increase of the product value is modeled by considering the successful placement of a new product on the market as well as the growing obsolescence of the product as a result of the competition’s innovations. A detailed description of the model can be found here.^11^

 Developing a new product is a highly dynamic and volatile process, which must be taken into account for any modeling scenarios. For this reason, it is crucial to consider the development and changes of all variables over time when evaluating the investment effectivity. Furthermore, interactions between the variables must be duly considered. The result can take the form of an overview of parameters, such as the one presented in Table, for the various scenarios considered in the company’s strategic decision-making.

**Table:** Variables for Chosen Strategic Decision in System Dynamics Models

**Scenario**	**Economic Result **	**Break-Even Point**	**Profitability**	**Total Number of Products Sold**	**Cumulative Sales **	**Relative Market Share at the End of Simulation**
Legislation old/new						
Foreign market entry (yes/no)						
New product (yes/no)						

## Conclusion

 What will, then, be the impact of regulation on innovation? The stakeholders are in agreement that this measure is necessary for the European Union to ensure greater patient safety and availability of information on products registered in the European Union. Manufacturers and innovators in particular often discuss the issue of readiness for this measure and express concerns regarding the clarity of the legislation and availability of the means to fulfil its requirements. Some critics of the legislation feel that the new regulation is overly complicated and ambiguous in some areas and are concerned about the related high costs and insufficient availability of notified bodies. Despite their frequent criticism of the new measures, the companies are working hard to adapt to the changes and meet the new conditions, while start-ups already take these conditions for granted. A clear answer will transpire in the coming years, showing on the economic results of companies, on the number of companies in the sector, and on the number of patents in the industry. However, I firmly believe that, as a result, new and safer medical devices will gain the advantage of quality over cheaper but poorer-quality competition, which will therefore lead to a development of the medical device industry.

## Ethical issues

 Not applicable.

## Competing interests

 Author declares that she has no competing interests.

## Author’s contribution

 PM is the single author of the paper.
